# Which factors affect post-transfer gaps in follow-up care? A qualitative study of the insights of healthcare providers in Sweden and Belgium

**DOI:** 10.1136/bmjopen-2023-079996

**Published:** 2024-08-17

**Authors:** Sandra Skogby, Eva Goossens, Bengt Johansson, Philip Moons, Ewa-Lena Bratt

**Affiliations:** 1 Institute of Health and Care Sciences, University of Gothenburg, Goteborg, Sweden; 2 Region Västra Götaland, Children’s Heart Center, Sahlgrenska University Hospital, Goteborg, Sweden; 3 Department of Public Health and Primary Care, KU Leuven, Leuven, Belgium; 4 Center for Research and Innovation in Care (CRIC), Faculty of Medicine and Health Sciences, Department of Nursing and Midwifery, University of Antwerp, Antwerp, Belgium; 5 Department of Surgical and Perioperative Sciences, Umeå Universitet, Umea, Sweden

**Keywords:** Adolescent, Congenital heart disease, Adult cardiology

## Abstract

**Abstract:**

**Background:**

Young people with congenital heart disease (CHD) are frequently affected by discontinued follow-up when transferring from paediatric to adult care. Identified predictors for discontinuation include mostly patient-related factors, and further knowledge of hospital and healthcare system factors is needed.

**Aim:**

This study aims to explore patient-related, hospital-related and healthcare system-related factors affecting continued follow-up care after transfer, as perceived and experienced by paediatric cardiology and adult CHD (ACHD) healthcare providers (HCPs) in Sweden and Belgium.

**Methods:**

This descriptive qualitative study included individual interviews with cardiologists, nurses and administrative staff, subjected to qualitative content analysis. A total of 30 HCPs from 13 specialist care outpatient clinics at 8 different centres in Sweden and Belgium were interviewed. HCPs were included if they had direct contact with patients and had at least 1 year of work experience.

**Findings:**

The findings illuminate three main categories of factors perceived by HCPs to affect continued follow-up care after transfer, including ‘care structure’, ‘care processes’ and ‘patient characteristics and circumstances’. Success was described as multifactorial, emphasising processes and structures of care, with a focus on collaboration, organisation, joint responsibility, resources, care relationships and transitional care interventions. Few differences appeared between paediatric and ACHD HCPs and between Swedish and Belgian HCPs.

**Conclusion:**

HCPs perceived factors on patient, hospital and healthcare system levels to influence continued follow-up. Process-related and structure-related aspects of care were perceived as more influential than individual patient characteristics. Hence, future research on discontinued follow-up care should focus on process-related and structure-related aspects of care delivery.

**Strengths and limitations of this study**

- Data collection from different hospitals and countries with different healthcare organisation models allowed for the exploration of hospital and healthcare system factors.
- Diversity in healthcare provider (HCP) characteristics is considered a strength, including cardiologists, nurses and administrative staff, providing a broader perspective.
- Interviews provided the opportunity for HCPs to elaborate on their local prerequisites and achievements.
- Only two countries were included in the study, the inclusion of more countries might have broadened the perspective.
- The fact that only university hospitals were included is considered a limitation.

## Introduction

 Today, the congenital heart disease (CHD) population is characterised by increased survival^1^ and fast-growing adult populations.^2^ Remaining challenges include providing appropriate follow-up care across the course of life,^3^ which is highly recommended to safeguard future health.^4^,^6^ Follow-up care not delivered within appropriate healthcare settings and/or timing could be considered discontinued.^7^ The transition towards adulthood in combination with chronic disease and transfer from paediatric to adult care makes young patients with CHD vulnerable to discontinuation. This vulnerability contributes to the numerous reports of young people with CHD experiencing discontinuation, with percentages ranging from 3.6% to 62.7%.^3^ Patient-related factors such as sex or CHD complexity have been shown to be associated with discontinuation.^8 9^ However, hospital and healthcare system factors likely affect discontinuation since the percentages differ across hospitals and regions.^10 11^ Discontinuation is further associated with adverse outcomes, including increased morbidity and the need for urgent (re)intervention,^12 13^ and therefore warrants prevention to ensure future health and reduce healthcare costs.

A prior multicentre study investigating discontinuation in young patients with CHD in Sweden reported low percentages of ‘no follow-up’. However, it also reported significant differences in percentages of ‘no follow-up’ between included hospitals.^10^ This could be considered an indicator of hospital-related factors’ relevance.^10^ However, this latter study could neither fully explain the differences between centres nor describe facilitating factors for the observed low percentages throughout seven hospitals in Sweden.^10^ To further increase understanding of influencing factors for continued follow-up, the perspective of young patients within the same cohort was inquired.^14^ To further scrutinise factors contributing to hospital differences, the perspectives of healthcare providers (HCPs) should be considered. Previous studies on HCP perspectives tend to focus on practices, attitudes and experiences of transfer and transition processes^15^,^19^ rather than specifically target factors perceived to affect continued follow-up care after the transfer.

This study aims to explore patient-related, hospital-related as well as healthcare system-related factors perceived to affect continued follow-up care after transfer among young people with CHD from the perspective of HCPs acting in paediatric cardiology and adult CHD (ACHD) outpatient clinics.

## Method

### Context

CHD lesions can be classified as either mild, moderate or severe. Patients with mild lesions require follow-up every 3–5 years, moderate lesions every 1–2 years and severe lesions every 6–12 months. Some patients are dismissed from follow-up during childhood, although the majority requires life-long follow-up care by specialised HCPs and a transfer from paediatric cardiology to adult healthcare services is required at varying levels of care depending on the severity of the lesion. Patient with complex CHD lesions require specialist care and patients with moderate or mild CHD lesions can be followed in shared care arrangements.^6 20 21^

Providing high-quality transitional care and facilitate continued follow-up care after transfer are challenging given the variation in disease complexity, follow-up intervals and transfer destinations as well as the individual patients’ self-care-management skills.

### Design

A qualitative descriptive design was applied to inquire HCPs’ perceptions of influential factors for continued follow-up care after transfer. Individual interviews in two sequences were performed and subsequently subjected to qualitative content analysis.^22^

To explore not only patient-related and hospital-related factors but also healthcare system factors, data were collected from two European countries. Sweden and Belgium apply different healthcare organisation models. The two countries provide representative example of European healthcare systems. In addition, Sweden and Belgium have previously reported relatively low percentages of discontinuation after transfer among young patients with CHD (6.6% and 7.3%, respectively).^8 10^ These low proportions make them suitable for investigation of facilitating factors for continued follow-up care after transfer, in addition to possible barriers.

### Setting

Nine tertiary hospitals from Sweden and Belgium were approached for participation, all comprising both paediatric cardiology and ACHD programmes. At the Swedish centres, transfer to adult care was standard practice at around 18 years of age, with no formal transition programme currently available. The Belgian centres transferred patients to adult care as standard practice around 16 years of age and one of the approached Belgian centres offered a formal transition programme at the time of data collection.

### Sample

The interview sample consisted of cardiologists, nurses and administrative staff working in paediatric cardiology or ACHD outpatient clinics at the hospitals approached. HCPs had to have direct contact with patients and at least 1 year of work experience to be eligible for inclusion. A convenience sample approach was applied, since the number of HCPs in each category could be limited at the included outpatient clinics.

### Data collection

Interview data were collected from 2021 to 2022 using two sequences, first ACHD HCPs and then paediatric cardiology HCPs. Eligible participants were approached after contact with the operative manager or equivalent. They were informed about the rationale and purpose of the study and were asked to provide verbal consent for participation. Two out of the nine approached ACHD clinics declined participation due to lack of time. Interviews were scheduled when convenient for the participants. Interviews were performed individually, digitally (or by telephone), and audio recorded. A semistructured interview guide was used (online supplemental material 1), and field notes were taken. The interview guide was evaluated after the first interview and considered satisfactory. Interviews lasted approximately 30–60 min. After analysis of ACHD HCP interviews, the interview guide was complemented and used for interviews with paediatric cardiology HCPs. Data saturation was considered attained after interviewing 30 HCPs from 13 clinics at 8 centres. No repeated interviews were performed. SS performed the interviews and had no prior relation to any of the participants.

### Analysis

Qualitative content analysis, as described by Graneheim and Lundman,^22^ was applied, with a combination of inductive and deductive approaches.^23^

First, ACHD HCP data were analysed inductively. Interviews were transcribed verbatim. Transcripts were not returned to participants for comments. Transcripts were read through repeatedly and sections of data addressing the study’s aim were retrieved as meaning units and subsequently condensed and coded. Codes were compared and clustered into descriptive categories and subcategories. To ensure trustworthiness, the process included repeated shifts between parts of and the complete data set, as well as discussions within the research group.^23^ Analysis of HCP data from paediatric cardiology was performed deductively^23^ to confirm the identified categories from ACHD HCPs and to add a paediatric cardiology perspective. Data were transcribed verbatim and read through, after which meaning units were identified and coded deductively into the subcategories identified in ACHD HCP data. Any remaining data would have been coded inductively.^23^

### Patient and public involvement

Patients or the public were not involved in the design, conduct, reporting, or dissemination plans of our research.

## Results

### Sample characteristics

In total, 30 HCPs participated in the interviews, including 13 (43.4%) cardiologists, 10 (33.3%) nurses and 7 (23.3%) administrative staff (table 1).

**Table 1:** Healthcare providers’ characteristics

	N (%)
Sweden	23 (76.7)
Belgium	7 (23.3)
Male	6 (20)
Female	24 (80)
Paediatric cardiology clinic	12 (40)
ACHD clinic	18 (60)
Paediatric cardiologists	6 (20)
Paediatric nurses	4 (13)
Paediatric administrative staff	2 (6.8)
ACHD cardiologists	7 (23.4)
ACHD nurses	6 (20)
ACHD administrative staff	5 (16.8)
Work experience abroad	9 (30)
Total	30 (100)

ACHDadult congenital heart disease

### Factors affecting continued follow-up care

**Figure 1:**
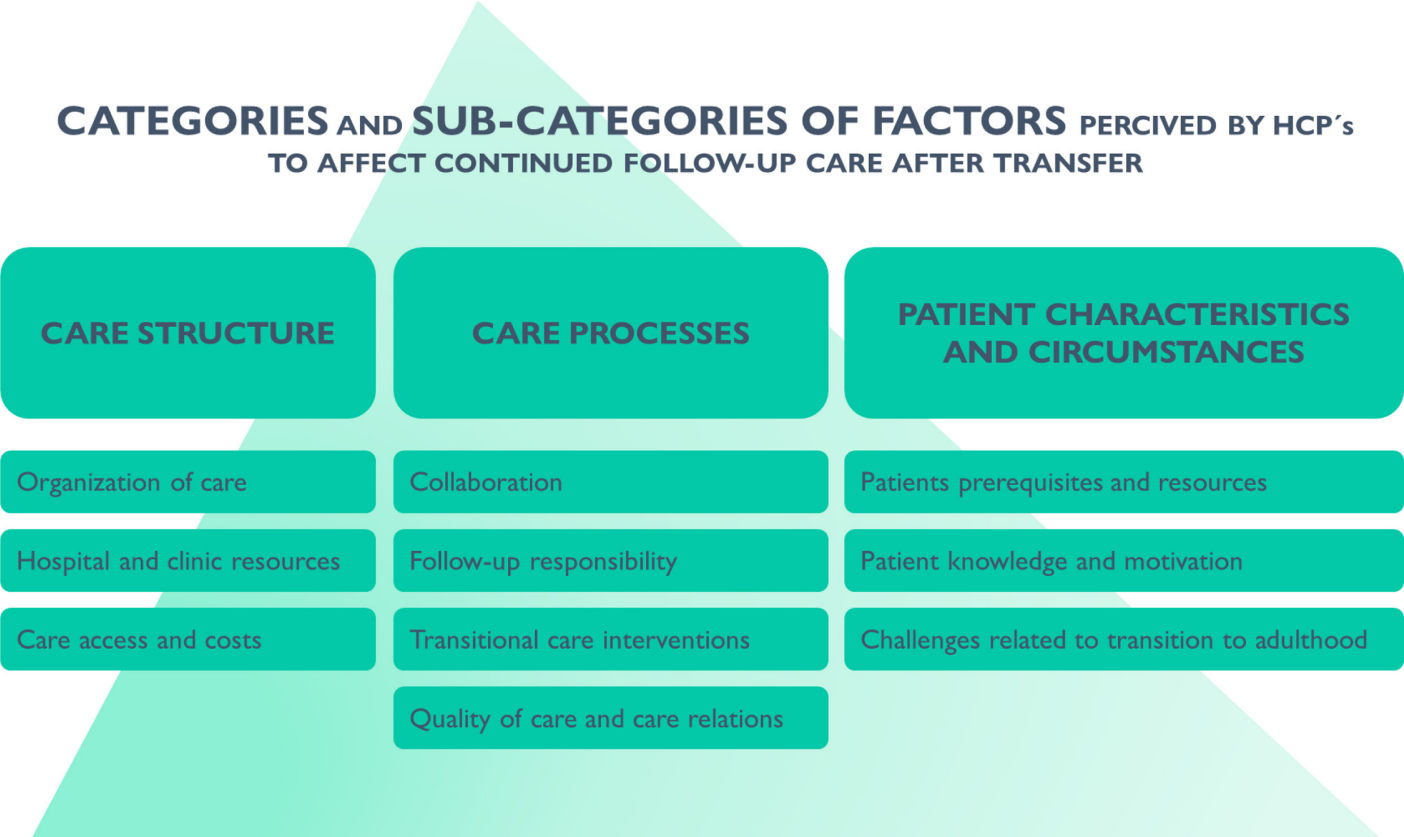
Overview of categories and subcategories from the qualitative analysis. HCP, healthcare provider.

**Figure 2:**
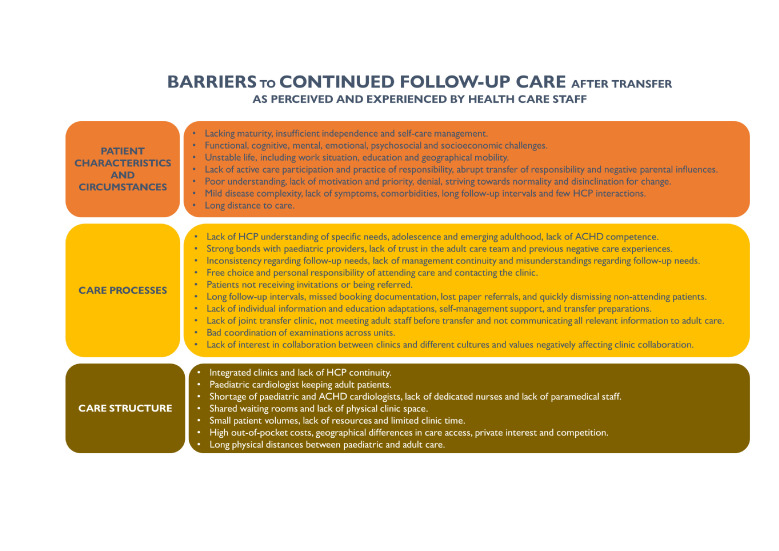
Summary of barriers to conducted follow-up care after transfer and experienced by healthcare staff. ACHD, adult congenital heart disease; HCP, healthcare provider.

Three main categories of factors emerged: ‘care structure’, ‘care processes’ and ‘patient characteristics and circumstances’. Appurtenant subcategories are stated in figure 1. Facilitators and barriers are briefly summarised in figures2  3.

**Figure 3:**
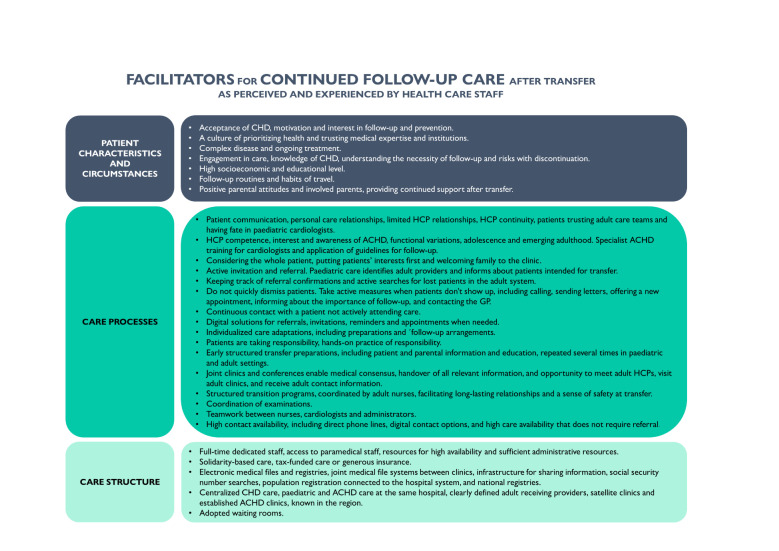
Summary of facilitators for continued follow-up care after transfer as perceived and experienced by healthcare staff. ACHD, adult congenital heart disease; HCP, healthcare provider.

Processes of care and care structure, rather than patients’ prerequisites and resources, were perceived to explain differences in discontinuation between hospitals and countries. Particularly highlighted success factors included preparations before the transfer, structured transfer routines, collaboration between paediatric and adult care, transitional care interventions, personal care relationships, full-time dedicated staff, sufficient administrative resources and active measures to keep patients in care.

### Patient characteristics and circumstances

Individual characteristics and circumstances of patients were perceived to affect continued follow-up, including challenges of transition, personal motivation and individual resources.

#### Challenges related to transition to adulthood

Lack of independence and varying maturity were described as possible barriers to continuing care after transfer. HCPs described how young people’s priorities differed from that of adults (Quote 1, online supplemental table 1), and more appealing aspects of life could be prioritised over follow-up. Patients were perceived to return to care later in life when they were more settled. Illness identity was raised as a possible barrier, as young patients could neglect their CHD while striving to be considered normal (Quote 2, online supplemental table 1). Geographical mobility, including relocation for work or education, or not living at registered addresses was other potential barriers, resulting in missed invitations or referrals. Temporary employment or being paid by the hour were additional barriers perceived by HCPs.

#### Patient knowledge and motivation

Patient motivation for follow-up was described as a protective factor. Patients who are in denial or feel uncompromised by CHD could have reduced motivation to continue care after transfer. However, acceptance and interest in prevention could increase motivation. Being exposed to cultures that prioritise health and trust medical expertise was described as protective. Motivation was considered important to attain knowledge of your disease, and this in turn facilitated acceptance. Understanding the necessity of follow-up was considered protective, while not understanding the risks of discontinuation was considered a risk factor (Quotes 3 and 4, online supplemental table 1).

#### Patient prerequisites and resources

Patients with complex CHD were perceived to receive more information and preparation before transfer and more structured transfers, including communication between paediatric and adult care. Higher disease complexity was considered protective, since symptoms or ongoing treatment could motivate patients and HCPs to become more alert, meaning complex patients were often prioritised. Milder disease complexity and a lack of symptoms could indicate to patients that follow-up was unnecessary, leading to them having fewer appointments and being minimally prepared for transfer, as well as having a poorer understanding of their CHD. This was therefore perceived as a risk factor (Quote 5, online supplemental table 1).

Functional variation, such as neuropsychiatric or cognitive disabilities, was considered a barrier, increasing the need for social, parental and HCP support and negatively affecting motivation, independence and self-care management. Psychosocial or socioeconomic factors could also influence follow-up, for example, stress, economic situation or language deficits. Since such aspects could make HCP communication and information processing more difficult. Long travel distances were also a possible barrier, requiring longer absence from work/school, increased travel costs or requiring access to own transportation.

Parental attitudes towards follow-up care and transfer could affect patients’ attitudes, behaviours and motivation (Quote 6, online supplemental table 1). To facilitate independence, patients had to practice responsibility and be engaged in their care. Abrupt transfer of responsibility was perceived as a risk factor and continued parental engagement after transfer could be protective.

### Care processes

Processes of care were perceived to affect continued follow-up care. Quality of the care delivery and care relationships were highlighted, as well as care responsibilities, transitional care interventions and collaborations.

#### Quality of care and care relations

Medical continuity was considered protective, and deviations from medical plans after transfer were deemed a risk factor. Applying follow-up guidelines was considered important to avoid incorrect dismissal. Verbal misunderstandings regarding follow-up needs were highlighted, and expressions such as ‘healthy’ could be interpreted by patients as meaning they did not need follow-up (Quote 7, online supplemental table 1). Specialist ACHD training for cardiologists was considered protective. Lack of ACHD competence, as well as adolescence and emerging adulthood competencies, could reduce HCP engagement and negatively affect interventions to keep patients in follow-up, such as outreach to non-attending patients (Quote 8, online supplemental table 1). ACHD clinics were considered especially aware and attentive to young people’s needs. Non-ACHD clinics could be less understanding of forgetfulness, missed appointments or geographical mobility (Quote 8, online supplemental table 1).

Personal care relationships, patient communication and patients’ trust in the care team were considered protective, increasing motivation and facilitating individualised care adaptation, such as planning follow-up together with the patient. Good treatment from HCPs was deemed to be facilitating, including an understanding of the young people’s situation, welcoming family to attend visits, allocating sufficient time, considering the whole individual and putting patients’ interests first. However, strong bonds between patients and paediatric HCPs could be a barrier, with patients and parents dreading changing HCPs and feeling a lack of trust in adult HCPs (Quote 9, online supplemental table 1). Nevertheless, a strong bond with paediatric HCPs could also lead to faith in the paediatric cardiologists’ decision to transfer care. Meeting adult HCPs before the transfer could increase trust and motivation. Long follow-up intervals could be a risk factor, making relationship building with HCPs more difficult.

#### Follow-up responsibility

Patients’ free choice regarding attending adult care and their newfound personal responsibility in attending care could be risk factors, although some HCPs argued that patients becoming independent and assuming responsibility could prevent discontinuation. HCPs could argue that patients are expected to attend follow-up, but this HCP mindset was considered a risk factor since many young patients require self-care management support.

Clear adult destination with active invitation and referral was considered protective, while a lack of referral was considered a risk factor, brought on by human error. Clear referral routines and keeping track of referral confirmations could reduce risks. Protective administrative processes included system searches for ‘lost’ patients and crosschecking to see whether patients were referred and attended care (Quote 10, online supplemental table 1). Risk factors in administrative processes were highlighted, such as missed booking documentation. To prevent missed invitations and referrals, paediatric care could inform adult clinics about patients intended for transfer, and these patients could be registered as awaiting referral. Patients who temporarily moved abroad could be kept on waiting lists, with annual clinic-initiated contact. Sending annual letters to patients to reinform them about upcoming visits and the importance of follow-up could be facilitating. Contactability was deemed protective, including direct phone lines for patients. Digital solutions were also highlighted, including digital contact options, digital invitations, text message reminders, digital referrals and digital appointments when needed. Receiving contact information for adult care before transfer was considered protective. In the case of missed appointments, active support from the clinic was considered protective, such as calling patients (Quote 11, online supplemental table 1), offering new appointments, cardiologists sending encouraging letters and not easily dismissing patients.

Paediatric cardiology was considered responsible for preparing patients for transfer, informing about the importance of follow-up, identifying suitable adult provider(s) and actively referring patients. Adult care was responsible for patients attending adult appointments and building trusting relationships. Adult care was also deemed responsible for continuing transition processes towards independence and acknowledging the need for extra support and care adaptations.

#### Transitional care interventions

Preparations and structures for transition and transfer were highlighted as protective features. Preparation should start early, ideally around 12 years of age. Information and education about CHD, self-care management, the importance of follow-up and risks associated with discontinuation were emphasised as protective. Areas of interest to patients, aspects of adolescence and adult life and information about adult care were also deemed to be priorities (Quotes 12 and 13 online supplemental table 1). It was felt that the quality of available information and education varied, but HCP continuity and individual adaptations were protective, particularly for patients with functional or cognitive challenges. HCPs should identify and address the need for support early, for example, by adapting the information and initiating relevant examinations or societal interventions to support future independence. Not adapting care was considered unethical and contributed to unequal care. In addition, trying to understand patients’ and parents’ perspectives on transition and transfer was considered protective, and here paramedical staff played an important role. Hands-on practice of independence and responsibility, for example, by attending visits without parents, was considered protective. Attaining knowledge and independence was considered a process, as not all patients had interest or motivation during adolescence. In addition, it was felt that adult HCPs should continue transitional interventions in adult settings. The same information that repeated several times, in both paediatric and adult settings, was deemed necessary to achieve knowledge (Quote 14, online supplemental table 1). Established contact between patients and adult HCPs and proper handover, including both medical and social aspects, were protective factors.

Transfer conferences and transfer clinics between paediatric and adult HCPs were considered facilitating. Transfer conferences for all patients, regardless of complexity, contributed to medical consensus. Joint transfer clinics provided an opportunity to meet adult staff and were perceived to increase trust. Lack of joint transfer clinic was often due to a lack of HCP interest or priority and considered a barrier (Quote 15, online supplemental table 1). Visiting the adult clinic could increase the young person’s sense of security and ease orientation on their first visit. Handovers between other professions, such as nurses and psychologists, could reduce the gap in psychosocial care and be protective.

Structured transition programmes were considered facilitating and having nurses coordinate the programmes was considered effective. Having adult CHD nurses function as transition co-ordinators was perceived to serve as a bridge between paediatric and adult care. Transition consultations with adult nurses enabled building relationships which would continue after the transfer, providing a sense of security (Quote 16, online supplemental table 1). During transition consultations, patients could receive education and information and practise taking an active part and speaking for themselves. Preparations were also facilitating for parents since their attitudes could influence the adolescents (Quote 17, online supplemental table 1). Helping parents to feel motivated about transfer was not only protective but it also supported the gradual transfer of responsibility—a process seldom completed at transfer.

#### Collaboration

Collaboration within and across clinics and hospitals was considered facilitating, especially between paediatric and adult care (Quote 18, online supplemental table 1). Collaboration could bridge gaps between providers and facilitate the implementation of structured routines and transition programmes. Collaboration between paediatric and adult care could facilitate knowledge exchange, for example, through joint transfer clinics, conferences or joint professional development activities. Collaboration between different ACHD clinics and collaboration between ACHD clinics and regional hospitals were considered protective, facilitating knowledge, awareness and inspiration. National registries and centralisation of CHD surgery contributed to collaboration and networking. Collaboration was considered time consuming, dependent on mutual interest and affected by individual HCPs and personal relationships between HCPs. Different hospital and clinic cultures, climates and values could complicate collaboration (Quote 19, online supplemental table 1).

Coordination of examinations across hospital units, for example, between the ACHD clinic and imaging department, was highlighted as protective. Poor co-ordination contributed to longer waiting times and more appointments, requiring more time or days off work or school, increased patient costs and decreased motivation.

Teamwork was highlighted as protective. Collaboration between nurses and cardiologists made it easier to identify special needs and contributed to patients feeling safe. A lack of dedicated nurses was a barrier, increasing the burden on cardiologists and increasing the risk of missing educational, psychosocial, emotional or cognitive issues and needs. Close collaboration between administrative staff, nurses and cardiologists was highlighted as important to keep patients in follow-up.

### Care structure

Factors at a hospital and healthcare system level, including care access, costs, organisation of care and clinic resources, were highlighted as affecting continued follow-up.

#### Care access and costs

A clear healthcare structure with clearly defined providers could be protective, while geographical differences in care access could be a barrier. Tax-funded and solidarity-based healthcare systems were considered to facilitate continued follow-up, reducing the impact of socioeconomic backgrounds (Quotes 20 and 21, online supplemental table 1). High healthcare costs were considered a barrier and generous insurance, or tax-funded care, contributed to low out-of-pocket costs (Quote 22, online supplemental table 1).

#### Organisation of care

Centralisation of CHD care was considered protective, whereas lack of centralisation was deemed to increase the risks of inappropriate care. Longer distances between paediatric and adult clinics or separate buildings required relocation of care provision and were considered a risk factor (Quotes 23 and 24, online supplemental table 1). Different hospitals for paediatric and adult care could complicate communication and information transfer. Infrastructure for information transfer across hospitals was important. Having joint medical file systems eased information transfer. Long intervals between the last paediatric and first adult visit were perceived as a risk factor, considering the vast changes in life during emerging adulthood.

Established ACHD organisations with follow-up care programmes were deemed facilitating. Smaller clinics could have insufficient resources and smaller patient volumes, which could lead to reduced competence. Having HCPs dedicated to ACHD care full time was considered protective, including nurses, cardiologists and administrative staff. Integrated units might generate part-time positions and require HCP to combine ACHD with other patient groups, leading to reduced ACHD competence and understanding of specific needs. For cardiologists and nurses, constantly switching between different patient groups could affect quality of care and lead to patients feeling less comfortable (Quote 25, online supplemental table 1). Paediatric cardiologists providing follow-up for adult patients was considered a risk factor for future discontinuation since ACHD competence could be lacking. Satellite clinics and shared care arrangements could be protective, reducing patient travel and shared burden between clinics. Awareness about ACHD clinics in the local region was facilitating. It was felt that ACHD clinics should make themselves visible and inform local hospitals about their services. Commercial or societal campaigns were perceived to increase awareness among patients and facilitate follow-up.

#### Hospital and clinic resources

Insufficient cardiologist resources negatively affected waiting times and appointment options and were considered a barrier. Being able to reschedule appointments and appointment options outside of office hours could facilitate follow-up. Having dedicated ACHD nurses was considered protective, as well as the resources to be accessible by phone, individualise care, provide extra support and work actively with transfer and transition. In general, larger hospitals were considered to have more resources than smaller hospitals and paediatric care to have more resources than adult care, creating potential quality gaps for patients at transfer. Sufficient administrative staffing resources provided structure for invitations, appointments and referrals. A limited number of patients per administrative staff were considered facilitating in getting an overview and being able to systematically identify ‘lost’ patients (Quote 26, online supplemental table 1). Administrative tools and resources were highlighted, including medical files, hospital databases, national registries, population registration and social security numbers, which provided an overview and enabled searches for lost patients.

Access to paramedical staff, imaging and interventions were considered protective. Lack of physical space, such as examination rooms and old worn-out facilities, could make patients less keen to attend care. Waiting rooms customised for young people were facilitating, while shared waiting rooms with other patient groups were deemed a barrier.

### Similarities and differences across paediatric and adult HCPs and countries

Paediatric and adult HCPs described no conflicting factors for (dis)continued follow-up. However, descriptions, emphasis or perspectives differed. Below follows a few examples. Both paediatric and ACHD HCPs emphasised collaboration between paediatric and adult settings and teamwork. ACHD HCPs more clearly emphasised the importance of the administrative staff and administrative resources. Both paediatric and ACHD HCPs highlighted the importance of actively initiating follow-up and preventing discontinuation. However, paediatric HCPs raised concerns about the level of support and active prevention in adult settings in general. In contrast, ACHD HCPs described their level of support and active prevention as more extensive than in non-ACHD settings. Both groups of HCPs highlighted structured preparations for transfer and transition programmes, but the paediatric HCPs more clearly emphasised the importance of individualised information/education and continued and repeated information/education in the adult settings. Consequently, having dedicated staff for this was highlighted. However, it was mainly only ACHD HCPs who raised issues about integrated clinics. Long follow-up intervals between paediatric and adult settings were raised as an issue by both groups, although patients remaining with paediatric cardiologists and the facilitating effect of satellite clinics were mainly raised as issues by ACHD HCPs.

No conflicting factors were highlighted by Swedish versus Belgian HCPs. However, different perspectives on some factors were noted. For example, regarding the healthcare system and costs, both Belgian and Swedish HCPs described the low cost and accessible care as facilitating. In this context, Swedish HCPs highlighted tax-funded and solidarity-based healthcare delivery, while Belgian HCPs highlighted generous healthcare insurance generating less barriers to CHD care.

## Discussion

This study has identified several factors perceived by HCPs to affect continued follow-up care after transfer in young people with CHD. These are factors which most probably interact, creating synergistic effects, and therefore should not only be considered separately but also jointly.

From an overall perspective, the identified factors share many attributes with previous research, which could be considered to strengthen the findings. Examples are similarities with previous reports on patient-related factors,^9 11 24 25^ similarities with factors described by young patients from the same Swedish centres^14^ and previous reports of HCP attitudes and experiences of transfer and transition.^15^,^19^

HCPs described factors that deemed to explain previously reported hospital differences in proportions of no follow-up,^10^ including processes of care and care structures, rather than patients’ prerequisites and resources. One facilitator highlighted was collaboration between paediatric and adult care, which enabled safe information transfer and structured transitional care interventions and contributed to knowledge exchange, increased patient trust and reduced risk of administrative mistakes at transfer. Collaboration has previously been highlighted as facilitating transfer and transition by HCPs.^17^,^19^ Participants in the present study described collaboration as depending on mutual interests and affected by conflicting values and cultures. Cultural differences between paediatric and adult centres have previously been described by Irish CHD HCPs as complicating transfer.^18^ Cultural differences can be difficult to bridge but are probably best tackled through interaction, understanding and collaboration. Distance between paediatric and adult clinics was another possible barrier, which is in line with a previous report on European cardiologists’ attitudes towards transfer, where adult and paediatric care within the same institution was considered facilitating for transfer.^17^ Geographical location can be difficult to do anything about, but increased collaboration could probably reduce some effects of distance.

A prior Swedish multicentre study including the same Swedish centres reported paediatric outpatient volumes as predictive for continued follow-up after transfer.^10^ However, it is not clear if outpatient volumes themselves affected follow-up; more probably, volumes affect aspects such as staffing resources or HCP competencies. Indeed, HCPs in the present study raised small patient volumes as a barrier affecting competence and resources and thereby continued follow-up. Clinic structure and resources were also highlighted, including the perceived barrier of integrated clinics, the facilitating effects of full-time dedicated staff and the barrier of insufficient administrative staffing resources. The findings could strengthen the hypothesis that the previously reported predictive outpatient volumes^10^ might have influenced clinic organisation, for example, in terms of full-time dedicated staff, administrative resources or separate or integrated clinics.^10^

HCPs taking active responsibility for follow-up was facilitating, for example, through active invitation, reminders and action when patients do not show up. This is in line with a previous investigation of perceptions and experiences of patients from the same Swedish centres, where young patients stated active invitation, reminders and extra support when missing appointments as strongly facilitating for continued follow-up.^14^ Administrative errors were highlighted in the present study as barriers and system searches for lost patients as protective. In case of missed invitations, it becomes the responsibility of patients to contact their clinic, which was considered a barrier. This is in line with the descriptions of young Swedish patients, for whom high trust in care and the expectation to be summoned could lead to long gaps in care after transfer.^14^ Varying maturity and independence at transfer described by HCPs in the present study, along with previous descriptions from patients needing support in attending care after transfer,^14^ emphasise the relevance of the HCPs described efforts to find lost patients in the system and their active measures to get patients to clinic. Lack of support from adult care has previously been highlighted as a barrier to transfer by Swedish HCPs but this is more emphasised in regional rather than university settings.^19^

Previous studies have indicated that structured transition programmes might positively affect discontinuation of care in terms of time intervals between visits^26 27^ and loss to follow-up rates,^28^ but further evidence is needed. The participants in this study highlighted transitional care interventions as protective, including, for example, joint transfer clinics, structured information and education and structured transition programmes continuing in adult care, which would ideally be coordinated by adult staff to bridge care during transfer. Similarly, in the previous study on young Swedish patients’ perceptions of factors affecting continued follow-up, aspects which could be related to transitional needs were highlighted as facilitating, such as meeting adult staff before transfer, interactions with HCPs about CHD and receiving information about follow-up needs and risks of discontinuation.^14^ Based on these studies, there seems to be an agreement between patients and HCPs that transitional care interventions before transfer facilitates continued follow-up care after transfer.

Healthcare system factors affecting discontinuation have rarely been described. However, regional differences in discontinuation rates have been reported in Europe, Canada and the USA,^3^ indicating an influence of healthcare system factors. Belgium and Sweden apply different healthcare organisation models but still highlighted similar system-related factors in this study, although from different perspectives. For example, possible barriers were competition or private practices, and facilitators were low out-of-pocket costs and centralised care. HCPs in this study perceived some healthcare system factors to be of relevance, but generally processes of care were emphasised as more influential than characteristics of the healthcare system itself.

### Methodological considerations

The main strength of the present study is the heterogeneity of perspectives, including both paediatric and adult HCPs from different professions, hospitals and countries, as well as different types of transitional care strategies. However, only two countries were included in the present study. Even though the two countries provide a good representation of European healthcare systems, there are always differences across countries. Inclusion of more or other countries might have affected the identified factors.

As only HCPs working at university hospitals were included, the findings lack a regional hospital perspective, which should be considered a limitation. Many patients are provided for in regional hospitals and their organisational structure is likely to differ from that of tertiary university hospitals.

The convenience sample approach could be considered a limitation for the present study; however, due to the limited number of eligible informants, it was considered the most appropriate approach.

## Conclusions

Factors related to *care structures*, *care processes* and *patients’ characteristics and circumstances* were perceived by HCPs to affect continued follow-up care after transfer. Success was described as multifactorial, emphasising processes and structure of care with a focus on *collaboration, organisation of care, joint responsibility, sufficient resources, personal care relationships* and *transitional care interventions*. There seems to be a large agreement between these HCPs’ perceptions of factors affecting continued follow-up care and previous reports from young patients with CHD. Process-related and structure-related aspects of care were emphasised as more influential for continued follow-up after transfer than characteristics of the healthcare system itself or individual patient characteristics.

## supplementary material

10.1136/bmjopen-2023-079996 online supplemental material 1

10.1136/bmjopen-2023-079996 online supplemental table 1

## Data Availability

Data are available upon reasonable request.
