# Supplementary material for: Which factors affect post-transfer gaps in follow-up care? A qualitative study of the insights of healthcare providers in Sweden and Belgium
Source: BMJ Open. 2024 Aug 17;14(8):e079996. doi: 10.1136/bmjopen-2023-079996 (PMC11331846; doi:10.1136/bmjopen-2023-079996)
Supplement: online supplemental material 1 [file bmjopen-14-8-s001.pdf]

Interview guide

<table>
<tr><td colspan="2">The overall aim is to explore factors associated with (dis)continued follow-up care after transfer in young people with CHD from a multicentre and international perspective.

Specific aims:
(i) to describe HCP´s perceptions and experiences of factors affecting continued follow-up care after transfer.</td></tr>
<tr><th>Research questions</th><th>Interview questions</th></tr>
<tr><td>Which factors do HCP´s consider as relevant for continued follow-up care after transfer?</td><td>We know from previous studies from various countries that many young patients with congenital heart disease do not attend their outpatient visits after they have been transferred from paediatric to adult care facilities.
- What are your thoughts about this in general?
Why do you think that is?</td></tr>
<tr><td rowspan="4">Which barriers and facilitators for continued follow-up care do HCP´s acknowledge or reflect upon at their own clinic/hospital?</td><td>Based on your experiences, what do you think could affect whether young patients attend follow-up visits or not after transfer?</td></tr>
<tr><td>Can you see or reflect upon any possible barriers for young patients to continue follow-up after transfer related to your own clinic or your own hospital?</td></tr>
<tr><td>Can you see or reflect upon any facilitating factors for continued follow-up for young people at your clinic or your hospital?</td></tr>
<tr><td>Is there anything that your clinic/hospital could do differently to get more young people to continue follow-up after transfer?</td></tr>
<tr><td rowspan="4">Which barriers and facilitators for continued follow-up care do HCP´s acknowledge or reflect upon within their own health care system as a whole?</td><td>We have seen differences between centres in terms of how many patients fail to attend a first visit after transfer,
- What are your thoughts on this difference?
- Why do you think that is?</td></tr>
<tr><td>We have seen that in Sweden/Belgium, more patients attend their follow-up visits compared to many other countries.
- What are your thoughts about this?</td></tr>
<tr><td>Can you see or reflect upon any possible facilitating factors for young patients to attend follow-up in your healthcare system as a whole?</td></tr>
<tr><td>Can you see or reflect upon any barriers for young patients to attend follow-up in your healthcare system as a whole?</td></tr>
<tr><td>Does collaboration between pediatric and adult care and the view on responsibility play a role for continued follow-up care?</td><td>What is your opinion about the allocation of responsibility between the pediatric and adult clinic to improve the transition and transfer to adult care?</td></tr>
</table>
