# Supplementary material for: Which factors affect post-transfer gaps in follow-up care? A qualitative study of the insights of healthcare providers in Sweden and Belgium
Source: BMJ Open. 2024 Aug 17;14(8):e079996. doi: 10.1136/bmjopen-2023-079996 (PMC11331846; doi:10.1136/bmjopen-2023-079996)
Supplement: online supplemental table 1 [file bmjopen-14-8-s002.pdf]

SUPPLEMENTARY TABEL 1. PARTICIPANT QUOTES

SUBCATEGORY	QUOTE NO.	PARTICIPANT	QUOTE
**Challenges related to transition to adulthood**	**1**	1<br>ACHD HCP	I think older patients consider health more important than young people do…. I would say that they generally are a difficult group to summon because they have so many reasons why they can't come…a lot of late cancellations…. because of…. sometimes very…. strange…. reasons…one could think that they should prioritize themselves a bit more…
	**2**	2<br>ACHD HCP	I know that many…some…don't want to think about their heart defect….most of the time it is a small aspect of their lives, there and then….having to be chronically ill, it might discourage…to know…that you have to be monitored your whole life…such a thought…might make them think…that you want to be like everyone else in society, and not require check-ups…
**Patient knowledge and motivation**	**3**	3<br>Paediatric cardiology HCP	I think that they should have a lot of knowledge about their disease and also knowledge about how the disease behaves and that we….to know that we often monitor people who feel and should feel completely healthy and that indication for intervention sometimes might appear long before you experience symptoms so that you know what we can assist with and see through ECG and echocardiography and physical examination, it contributes to the check-up, it is not just about how you experience your body.
	**4**	4<br>Paediatric cardiology HCP	When you look back at patients who were lost from paediatric cardiology, I feel that they probably did not receive adequate information as a patient, that they probably had not understood the heart condition and surgeries they had, why it is important with check-ups, and that it is important with follow-up…
**Patient prerequisites and resources**	**5**	5<br>Paediatric cardiology HCP	If you have a more complex and serious heart defect, then I don't think that you will be missed, because health care is very alert, I rather think that those who are a bit less and feel quite symptom-free and might come here every other year to paediatric cardiology…
	**6**	6<br>Paediatric cardiology HCP	I just wonder if it is only ….in the transfer that they get lost because….if we lose a patient in follow up it is very often these are also the patients that were not very faithful in childhood, so think their parents play a very important role in there to…if they…if they (..) a very good follow-up in childhood and they and their children know it is important, it is easier for the children and the youngsters to accept that it is necessary….
**Quality of care and care relations**	**7**	7<br>ACHD HCP	there is always a rupture when they become...when young people become adults and get to steer themselves…there I think…it happens that the paediatric cardiologist has said, that yes…or the surgeon ...that you are now cured from this disease and then they don't think that they require any follow-up…
	**8**	8<br>ACHD HCP	yes…. understanding, if you only look at our clinic, there is perhaps a bit more understanding…I think, that we understand this… I can compare with my colleagues who have general cardiology clinic…they are not used to talking about pregnancies and fertility, and if they work it is more about ..which…we discuss which jobs they can do, and what they can and cannot do…if you are at the other end of your working career, what you can continue with, what you can't do, there I think that we have a strength, that we perhaps have a greater understanding for these young people …and those challenges…it is not unique for us but …..then I think that we have an advantage that…./……../.. yes I think we might have a greater understanding for the fact that you don't live at your registered address…that you…when you are 21….you move…when you are 19 or 20 you might move away from home, but you don't change your registered address…you start school…you are travelling….you don't think about the fact that you have to be registered and that we send out invitations….or they don't come because they did not receive the invitation, because it is at mommy and daddies…or waiting on it….sometimes we might be a bit late with the invitation, that you send it 2 or 3 weeks before…I think there is…I think that an ACHD clinic might have a greater understanding that these things don't work…
	**9**	9<br>ACHD HCP	I just think that people don't like change…they are used to a certain doctor or a certain department and they have been building up a relationship for several years... with that department or with that specific doctor and some people find it hard to just give it up and give their trust into the hands of someone else… they are afraid that their file will not be known as well as… as they were used to at paediatric department... and I think that that's the main concern they have…(…) … of course the relationship with .. with the department...I mean we have several doctors working for the congenital heart disease department…and if people know that they are coming to one specific doctor and they have a lot of trust and confidence, then they will make the effort to come…. again, that the ...yeah that's a trust factor…if they have a good relationship over here with the nurse with the doctors ... they will be more motivated...to make it to the appointment…

**Follow-up responsibility**	**10**	1<br>ACHD HCP	… and these…it is those that I always go through, every month I always go through everyone who is booked at ACHD, they should have a follow-up and specifically for these transfer patients, I make sure that there is a referral from paediatric cardiology
	**11**	9<br>ACHD HCP	IP: also try yeah to be…eh…proactive and if a patient is yeah….we know some of our patients will not be so motivated to come the next year and then it is up to us to when we see that they are not showing up or that they miss their appointment it is up to us to contact them again and just don't let them go…be proactive and try to get the hospital……<br>I: and you think that this work that you do, this proactive work is keeping patients in follow-up?<br>IP: yeah some of them yes I am convinced yes if we would not call them they would not come …<br>I: okay….and do you think that the phone call is essential here or could you have the same effect by a new appointment by mail or?<br>IP: no by mail it is not working …no<br>I: why do you think the phone call is so essential?<br>IP: because they need to …yeah…they are confronted with someone directly…by phone by email…it is just easy to ignore…delete the email and it is gone ..by phone, of course, yeah they can choose not to pick up ..but then when we call anonymously ..they don't know who is calling and we get them on the phone anyway …so it is not so easy to just ignore us….
**Transitional care interventions**	**12**	10<br>ACHD HCP	The other thing is... and this is more scientific, I think... If you do not prepare the patients in an efficient way for the transfer, and if you do not stress on the fact that follow up is really needed… Yaa, patients will not, or parents of patients, will not see the benefit of continuous follow-up… and I think if centres do not stress that part… it is quite obvious then, patients decide to say, ok, paediatric cardiology is finished now, so for me, it's enough…and so I... I think everything is going well and, and... I do not feel the need for follow-up, so, and also my physician is not really pushing me into follow-up protocol.
	**13**	6<br>Paediatric cardiology HCP	it is our duty to inform our patients in time, that the paediatric period is just a time period and even if there is a very close relationship with paediatricians and the paediatric ward, it has to change when they get older because the problems change and the situation and the family changes so I think that is a major….one of our major duties together with informing patient about their pathology, the impact it has on their daily life, (..) part of medication, the importance of endocarditis prophylaxis…and a little bit depending on the maturity of patients I also talk about pregnancy, genetics these kind of things and…if they allow me the choice of the profession they choose for later because…(..)…type of (..)…some professions are just not very suitable …and I think it is very important that we make sure ..that our patient trusts and has faith in the adult cardiologist ….and that might be something which is overlooked…when patients are transferred to another team…so I think the responsibility of the paediatricians we have to take care that if we transfer our patients to the adult ward that all details are thee, not only the medical details, but also the psychological and social factors that are important in that family because what adult cardiologist tend to forget is that you have a long lasting relationship …
	**14**	6<br>Paediatric cardiology HCP	and I think that once they are at the adult ..the adult ward, the adult cardiologist…has stressed that paediatricians have given all the information that they needed at the time, but they need to repeat….because sometimes I have the impression that they think that we didn't explain anything at all…the patients forget, you have to repeat it over and over and over and over and over again….so they still need to continue, to repeat the information…because that often happens that they get the information again at the moment of transfer, but they don't get it again after five or after 10 years…so I think that's a thing that is very important for the adult, the adult ward, that they know that first years after transfer, they are still very young, and a lot of patients are not responsible enough to take care of all aspects of their treatment and their follow-up, so one should keep a close eye, especially in the beginning…and involving the parents in the beginning in helping their children to take their own health in their own hands….
	**15**	4<br>Paediatric cardiology HCP	Yes well, I think it is sad that the ACHD doctors don't want joint transfer clinics. They don't have time for that you see. Earlier we had a conference, one in the spring and one in the autumn, but now they don't have time for that either. So, I miss the interest from the adult side… I think that we have had frequent contact with our patients. We have had quite high continuity with our patients, we know our patients by heart, we know our patients by their given names and can recall their entire social context if you only say their given names. There is such a contrast when you arrive at the adult side and perhaps don't have that support and control. Just to have seen the face of the doctor who is going to follow you onwards or at least start to follow you onwards, I think is very important, but that interest does not exist at the ACHD unit.

	**16**	11<br>ACHD HCP	IP: yes, the joint meeting and I am going to the paediatric ward, and I am doing, I am making the bridge with them, together with the paediatric cardiologist…<br>I: do you think that it is important that you are going to paediatric care and not the other way around?<br>IP: yes…I discussed with the new transition coordinator for the hospital and mostly and also when you are reading papers about transition consultations, mostly it is a clinical nurse specialist from the paediatric ward… she or he is preparing the patients and giving them to the adult health care providers, but I think that advantage of our way of working is that…I am, I can, we can get to know each other and then they don't have to lose me…do you understand, and we can make the difference and I can talk with them about how we are working at the adult ward and we can talk about that and yeah I think…we.. you don't have to say goodbye again…
	**17**	12<br>ACHD HCP	it it's...we have been talking about patients.. I think in the process of transition the parents are key players as well so they also need to be ... well convinced of the fact that their child as they see it at that moment will need to transition and need to be transferred to adult facilities… in the way I see it with many patients, the young people themselves they.. most of them are quite okay with transitioning…if we see a barrier or it is not a barrier but if we see.. if we see some hesitation, it is mostly with the parents…so I think we we should really invest in ways to to yeah motivate parents to transfer their children and I know I mean I have children myself I ..they are beyond the transition phase obviously but I realize how difficult that must be yeah …and sometimes I talk about these aspects with with parents …they have seen their child so very sick and then they saw the paediatric cardiologist and they spent weeks some of them months in the hospital and were seeing this same person and all of a sudden you have to say goodbye so yeah…it is it is not easy
**Collaboration**	**18**	6<br>Paediatric cardiology HCP	but we are a very strong team, congenital cardiology, which means that there is a very close relationship between the paediatric cardiologists and the adult cardiologists, we meet every week together, and those who do the cats, those who do the catheter…the operations… are the same...so the only thing that changes when they go to the adult ward, is the cardiologist because the rest of the team remains the same…and our patients know that if they need some more treatment or a change in surgery, they are discussed in a team where everybody is there …paediatric cardiologist and the adult cardiologists, and the surgeon who has done the surgery before…. I think that really helps them…to know that they remain in the same team…<br>and I think that if a patient knows that it is only the cardiologist that changes …that the team for the rest remains the same, it is quite reassuring because they know that their file, the file is kept rigorously and the history is well known so I think that helps …eh… and probably also the relationship between the paediatric cardiologist and…it moves in both directions because I see that the patients with whom I have a very strong relationship, they find it more difficult to leave the paediatric ward,…but they know that if we send them somewhere else...it is because we care about them …and they know it is necessary…so I think that the …the organization of the system and of the the …the contact with the paediatric and the…faith the patient has in the paediatric cardiologist can make a big difference…
	**19**	6<br>Paediatric cardiology HCP	I hope I can say this, but I think there is a big difference in …the teambuilding between the paediatric cardiologists and the adult cardiologist in XX who really work as a team, we know each other personally, and we have the same, let's say values, when it comes to life and when it comes to treatment…and I know that in XX it is different…it is very different and they are to separate…separate units that have big difficulties to find each other
**Care access and costs**	**20**	4<br>Paediatric cardiology HCP	In part it is our tax-funded care, which is amazing, that is a success factor. That we have, we give care based on needs. And solidarity-based finances, that is absolutely a success factor.
	**21**	13<br>ACHD HCP	Yes, what I think everyone has…that is how it is in western Europe that all have access to care, regardless of who you are…and I think that the public financing makes …it is not about your background, you will get…on paper, everyone has the same opportunities, you are not limited for lacking insurance…or ….should we look at socioeconomics we would probably see that people with lower socioeconomic status don't get the same care, like it is in general, that probably applies to the ACHD population I would guess…but I think that most have access, are being offered and get access to the care that we have, that I think is an advantage….it is an advantage to have one principal health care provider in the country with information transfer and continuity…
	**22**	12<br>ACHD HCP	other factors I think other than that health care system, I think we are quite lucky in xx, most costs and most care is reimbursed, or I mean there are very low fees that patients need to pay … so I think that we are quite fortunate…
**Organization of care**	**23**	10<br>ACHD HCP	First of all, I think you have the logistics part. That means that… when…paediatric cardiologist decides to stop the follow up at the paediatric department... then the patient has to shift to the adult department, and I do know in some centres or in some countries… the distance between the paediatric part and the adult parties rather large areas… it's a long way to move from one site to the other. Sometimes it is also in another hospital, and sometimes its… maybe even in another country, you know. So, the thing is that, yeah, the logistics are not optimal. I think the risk is higher that…patients decide not to stay in follow-up, and then they go more to a local cardiologist or and if they feel well, then maybe they just get stuck with a general practitioner. You know... so I think this is based on a logistic part.

	**24**	6<br>Paediatric cardiology HCP	I think it depends a bit on how the system is working, I think if they have to move from one hospital to the other, it is much more difficult than if they stay in the same building I would say, or the same hospital… (….)… I think it is, it is not the same building, but they are very close to each other, so they don't need to change their routines in coming to the hospital… …… yeah, I think these are the main things…the positive factors that we have I think…
	**25**	13<br>ACHD HCP	IP: I only see my ACHD patients, I don't see other patients …...(..)…it is not a mix of ACHD patients and general cardiology patients, when I have an ACHD clinic, it is ACHD patients who attend.<br>I: yes…do you think it matters if you as a cardiologist see only CHD patients or other patients as well?<br>IP: yes it does….well I think that if you…I don't know how they arranged it at other places between…if you would have a clinic and suddenly a 78-year-old patient comes with atrial fibrillation and the next patient is a univentricular heart …I think you might lose focus a bit…focus on…now it is young patients, similar issues….we have prepared then with a nurse who takes blood pressure, survey and technician who does the eco, so for the logistic reasons, it is smooth to have it all at once…but to mix a general cardiology clinic, atrial fibrillation and heart failure with ACHD patients that…. I don't think will turn out well…because you lose focus…then you need to span over everything from treating atrial fibrillation in a 78-year-old or heart failure in a 69-year-old to talking about childbearing and pregnancy...I think you… I think it is difficult to jump from this to that when you have a clinic…. The first one wanted kids, the other one is afraid to die from age…. I think you …. I think you will be better if you focus a bit on one of them…. I think that's better….<br>I: do you think it could affect how many who choose to come and not to come?<br>IP: I do….I think that you might seem more engaged… or you are or you become more engaged if…if you are into what you talk about… so I think it is nice for patients to see a doctor who is engaged and knows what he is talking about….rather than….I think you …you don't see the same things….if you have heart failure in an old patient in your mind…you do have other symptoms as young patients and other issues of course…
**Hospital and clinic resources**	**26**	1<br>ACHD HCP	IP: yes, I believe so, absolutely…and I think it is a bit because….since I only work with this I can spend more time on cross-checking….if I think about the assistant nurses booking and summoning our other patients….they would never have the time nor the possibility, partly related to the amount, going through all patients and going through all waiting lists…there is no time for that….this is something which I have prioritized for us…you take the time…and I know that they are now working a bit towards assistant nurses summoning perhaps for specific doctors or patient groups…to get a better overview, since you learn about your patients…you know that….yes, with some there are no problems and with others, you have to spend more time….and I can often through a telephone call make it easier for the patient since I already….before I open the file….know what it is all about…but the others have such huge amounts….and I don't, I only have one patient group.<br>I: do you think that this could be a success factor for you?<br>IP: yes, I do…because we have reflected at our clinic, what makes us succeed so well, partly with the waiting list, the number of patients who don't get to come in time….and then how many you actually lose…and it has a lot to do with the searching, backtracking…that you find them again….and that time I don't think exists for the others…
