# Supplementary material for: Which factors affect post-transfer gaps in follow-up care? A qualitative study of the insights of healthcare providers in Sweden and Belgium
Source: BMJ Open. 2024 Aug 17;14(8):e079996. doi: 10.1136/bmjopen-2023-079996 (PMC11331846; doi:10.1136/bmjopen-2023-079996)
Supplement: Supplementary file 3 [file bmjopen-2023-079996.reviewer_comments.pdf]

PEER REVIEW HISTORY

BMJ Open publishes all reviews undertaken for accepted manuscripts. Reviewers are asked to complete a checklist review form and are provided with free text boxes to elaborate on their assessment. These free text comments are reproduced below.

ARTICLE DETAILS

**TITLE (PROVISIONAL)**	Which Factors Affect Post-Transfer Gaps in Follow-up Care? A Qualitative study of the Insights of Health Care Providers in Sweden and Belgium
**AUTHORS**	Skogby, Sandra; Goossens, Eva; Johansson, Bengt; Moons, Philip; Bratt, Ewa-Lena

VERSION 1 - REVIEW

**REVIEWER NAME**	Boongird, Atthaporn
**REVIEWER AFFILIATION**	Mahidol University
**REVIEWER CONFLICT OF INTEREST**	I have no conflict of interests about this review.
**DATE REVIEW RETURNED**	13-Jan-2024

**GENERAL COMMENTS**	Your manuscript has mentioned the real world healthcare of congenital heart disease from childhood to adult, which are different in two countries and can be more varieties all over the world. The studied is mainly narrative exploration of many caregivers, which is subjectively understandable. After review all of the contents, I have minor questions as the following.<br>1. What is the tragic incidence of discontinued follow up of this cardiac patient? Is there any reports that lost follow up causing significant morbidity and mortality. If this information is available, it will emphasize the necessity of the improvement of healthcare process with cost effectiveness.<br>2. This is a health care profession perspective. It is one way interview. Patient and family interviewing is not directly explored. The success of healthcare is two way communication to build up the optimal transfer follow up from child to adult.<br>3. As my understanding, the simple case and complex cases are judged by cardiologists, cardiac surgeon, which depends on guideline standard care. I suggest another diagram to see the whole pictures of case with different follow up care plans.<br>4.Is the patient registry can be transferred to different hospitals in the same country for this chronic congenital heart disease? This is an important for access healthcare anywhere safely.<br><br>Finally, this paper underscored the collaboration, organization of care and sufficient resources and transitional care plan to improved patient care plan from childhood to adulthood in many aspects. It is challenging problems in different health care providers in modern country and developing countries all over the world.

**REVIEWER NAME**	Bravo-Jaimes, Katia
**REVIEWER AFFILIATION**	Mayo Clinic in Florida
**REVIEWER CONFLICT OF INTEREST**	None

DATE REVIEW RETURNED	01-May-2024

GENERAL COMMENTS	Skogby et al present a qualitative study focused on factors that affect post-transfer follow-up care among young patients with congenital heart disease from 30 health care professionals' perspective working at 7 congenital centers in Belgium and Sweden. They performed qualitative interviews to both ACHD and pediatric cardiology health care professionals and identified patient, hospital and systemic factors that influenced transition and continued follow-up. From the patient perspective, they highlighted that competing priorities when becoming an adult, illness identity, knowledge, motivation and existing resources (socioeconomic, parental, etc) play a significant role in future follow-up. From a hospital or care process perspective, trust in an inclusive medical team, ACHD competence were seen as protective factors but strong bonds between pediatric health professionals and fear to change to an adult one was a deterring factor but can be mitigated by collaborative transition programs or joint transfer clinics. System-based factors included care access and costs, having ACHD nurses and administrative staff that can track lost patients. Whereas their findings are not novel, the qualitative approach used it is well appreciated and can help inform decision-makers. I would encourage the authors to clarify two aspects of this paper:<br>1. They highlighted that ACHD professionals were mainly the ones raising issues about integrated transition clinics or facilitating satellite clinics. Please elaborate further.<br>2. Despite the Belgian professionals were the only ones with a formal transition clinic starting at the age of 16, this did not arise as a conflicting factor. Please elaborate further.

VERSION 1 – AUTHOR RESPONSE

Reviewer: 1
Dr. Atthaporn Boongird, Mahidol University

Comments to the Author:

Dear authors

Your manuscript has mentioned the real world healthcare of congenital heart disease from childhood to adult, which are different in two countries and can be more varieties all over the world. The studied is mainly narrative exploration of many caregivers, which is subjectively understandable. After review all of the contents, I have minor questions as the following.

1. What is the tragic incidence of discontinued follow up of this cardiac patient? Is there any reports that lost follow up causing significant morbidity and mortality. If this information is available, it will emphasize the necessity of the improvement of healthcare process with cost effectiveness.

Thank you for this comment.

In this paper, we refer to discontinuation as "Follow-up care not delivered within appropriate healthcare settings and/or timing could be considered as discontinued" (1). The prevalence of discontinuation varied across previous reports. Among young people with CHD, previous reports range from 3.6% up to 62.7% (2). A plethora of factors can probably explain these broad variations. However, one of them could indeed be related to hospital and healthcare system characteristics since percentages of discontinuation have been shown to differ across both hospitals and regions (3, 4). Effects on mortality could be hypothesised and previous studies have reported an association between discontinuation and morbidity and the need for urgent interventions (5, 6). This could be considered a strong argument to ensure continued follow-up care from a cost-effectiveness perspective.

The above statements are provided in the introduction on page 4. To clarify this reasoning, we have added a sentence on the benefit of ensuring continued follow-up care from a healthcare cost perspective.

2. This is a health care profession perspective. It is one way interview. Patient and family interviewing is not directly explored. The success of healthcare is two way communication to build up the optimal transfer follow up from child to adult.

Thank you for these comments.

Indeed, both the perspectives of patients and healthcare providers can provide valuable information. The authors previously interviewed young patients with CHD in Sweden, investigating their perceptions of factors affecting continued follow-up care (7). Studies on young people's perceptions of the transition to adult care are known (8-10). The present study aims to broaden the perspective even further by also inquiring about the perspective of HCPs, as described in the rationale of this respective manuscript on page 4. The findings we have obtained in both this present study and the prior study on young patients (7), published in 2021, are also repeatedly contrasted in this paper's discussion section. Hence, we believe we address the reciprocal relationship between patients, families, and healthcare providers.

3. As my understanding, the simple case and complex cases are judged by cardiologists, cardiac surgeon, which depends on guideline standard care. I suggest another diagram to see the whole pictures of case with different follow up care plans.

Thank you for this comment.

Indeed, complexity and follow-up arrangements vary in the CHD population. We have added a section providing a description of the different follow-up plans for each respective level of CHD complexity based on the current set of guidelines. This information was added in a section describing the context as part of the method section.

4.Is the patient registry can be transferred to different hospitals in the same country for this chronic congenital heart disease? This is an important for access healthcare anywhere safely.

Thank you for this comment.

Indeed, having access to medical records from previous care givers can enhance patient safety. The access to medical patient records differs geographically across the centres in the present study. However, having an infrastructure for information transfer and/or joint medical file systems was raised

as a facilitating factor since it eased information transfer. This is described in the result section on page 15-16.

Finally, this paper underscored the collaboration, organization of care and sufficient resources and transitional care plan to improved patient care plan from childhood to adulthood in many aspects. It is challenging problems in different health care providers in modern country and developing countries all over the world.

Thank you for this comment.
We agree with this comment and hope that our paper contributes to the understanding of this issue.

Reviewer: 2
Dr. Katia Bravo-Jaimes, Mayo Clinic in Florida

Comments to the Author:

Skogby et al. present a qualitative study focused on factors that affect post-transfer follow-up care among young patients with congenital heart disease from 30 health care professionals' perspective working at 7 congenital centers in Belgium and Sweden. They performed qualitative interviews to both ACHD and pediatric cardiology health care professionals and identified patient, hospital and systemic factors that influenced transition and continued follow-up. From the patient perspective, they highlighted that competing priorities when becoming an adult, illness identity, knowledge, motivation and existing resources (socioeconomic, parental, etc) play a significant role in future follow-up. From a hospital or care process perspective, trust in an inclusive medical team, ACHD competence were seen as protective factors but strong bonds between pediatric health professionals and fear to change to an adult one was a deterring factor but can be mitigated by collaborative transition programs or joint transfer clinics. System-based factors included care access and costs, having ACHD nurses and administrative staff that can track lost patients. Whereas their findings are not novel, the qualitative approach used it is well appreciated and can help inform decision-makers. I would encourage the authors to clarify two aspects of this paper:

1. They highlighted that ACHD professionals were mainly the ones raising issues about integrated transition clinics or facilitating satellite clinics. Please elaborate further.

Thank you for this comment.

Indeed, the issue of having an integrated clinic, meaning a clinic where not only ACHD patients are cared for but also another heart-related diagnosis, such as heart failure or arrhythmias. The concern was related to the fact that HCPs needed to divide their time between ACHD patients and other conditions, which could impact competence and understanding of patient's specific needs. In contrast, being dedicated full-time to the patient group was considered facilitating. Indeed, this was mainly raised by ACHD HCPs and, to a lesser extent, by paediatric HPCs.

Also, the facilitating effect of satellite clinics was mainly addressed by ACHD HCPs. A possible explanation could be that this phenomenon is more common in adult care compared to paediatrics, but the present data do not strengthen such conclusions.

2. Despite the Belgian professionals were the only ones with a formal transition clinic starting at the age of 16, this did not arise as a conflicting factor. Please elaborate further.

Thank you for this comment.

Indeed, only one centre in Belgium provided a formal transition program, starting at the age of 16 years. The fact that the centre provided different types of care was considered a strength in this study since different experiences and perspectives can provide a more nuanced perspective on possible facilitators and barriers. This has been clarified on page 21. However, when reviewing the results obtained from the interviews, we do not consider this as a conflicting factor since both paediatric and adult care highlighted the facilitating effect of a formal transitional care strategy, as described on page 17.

In addition to the changes suggested by the reviewers. Minor changes in wording and spelling have been made to the manuscript.

References

1. Skogby S, Bratt E-L, Johansson B, Moons P, Goossens E. Discontinuation of follow-up care for young people with complex chronic conditions: conceptual definitions and operational components. BMC health services research. 2021;21(1):1343.
2. Moons P, Skogby S, Bratt EL, Zühlke L, Marelli A, Goossens E. Discontinuity of Cardiac Follow-Up in Young People With Congenital Heart Disease Transitioning to Adulthood: A Systematic Review and Meta-Analysis. Journal of the American Heart Association.
3. Skogby S, Moons P, Johansson B, Sunnegårdh J, Christersson C, Nagy E, et al. Outpatient volumes and medical staffing resources as predictors for continuity of follow-up care during transfer of adolescents with congenital heart disease. International journal of cardiology. 2020.
4. Gurvitz M, Valente AM, Broberg C, Cook S, Stout K, Kay J, et al. Prevalence and predictors of gaps in care among adult congenital heart disease patients: HEART-ACHD (The Health, Education, and Access Research Trial). Journal of the American College of Cardiology.
5. Cordina R, Nasir Ahmad S, Kotchetkova I, Eveborn G, Pressley L, Ayer J, et al. Management errors in adults with congenital heart disease: prevalence, sources, and consequences. European heart journal. 2018;39(12):982-9.
6. Yeung E, Kay J, Roosevelt GE, Brandon M, Yetman AT. Lapse of care as a predictor for morbidity in adults with congenital heart disease. International journal of cardiology. 2008;125(1):62-5.
7. Skogby S, Goossens E, Johansson B, Moons P, Bratt EL. Qualitative study of facilitators and barriers for continued follow-up care as perceived and experienced by young people with congenital heart disease in Sweden. BMJ open. 2021;11(10):e049556.
8. Burström Å, Bratt EL, Frenckner B, Nisell M, Hanséus K, Rydberg A, et al. Adolescents with congenital heart disease: their opinions about the preparation for transfer to adult care. European journal of pediatrics. 2017;176(7):881-9.
9. Burström Å, Acuña Mora M, Öjmyr-Joelsson M, Sparud-Lundin C, Rydberg A, Hanseus K, et al. Ready for Transfer to Adult Care? A Triadic Evaluation of Transition Readiness in Adolescents With Congenital Heart Disease and Their Parents. Journal of family nursing. 2019;25(3):447-68.
10. Burström Å, Öjmyr-Joelsson M, Bratt EL, Lundell B, Nisell M. Adolescents with Congenital Heart Disease and Their Parents: Needs before Transfer to Adult Care. Journal of Cardiovascular Nursing. 2016;31(5):399-404.

VERSION 2 - REVIEW

**REVIEWER NAME**	Bravo-Jaime, Katia
**REVIEWER AFFILIATION**	Mayo Clinic in Florida
**REVIEWER CONFLICT OF INTEREST**	None
**DATE REVIEW RETURNED**	02-Jul-2024

**GENERAL COMMENTS**	The authors have addressed the prior topics raised.
